# FCRL1 and BAFF mRNA Expression as Novel Diagnostic and Prognostic Biomarkers in Diffuse Large B-Cell Lymphoma: Expression Signatures Predict R-CHOP Therapy Response and Survival

**DOI:** 10.3390/ijms26031269

**Published:** 2025-01-31

**Authors:** Hiba S. Al-Amodi, Hanan M. Bedair, Suzy Gohar, Dalia Abdel-Wahab Mohamed, Eman M. Abd El Gayed, Mahmoud Nazih, Sahar Badr Hassan, Eman S. Sawan, Esraa Elsayed Elmahdy, Asmaa Mosbeh, Alaa Efat, Shimaa Abdelsattar

**Affiliations:** 1 Biochemistry Department, Faculty of Medicine, Umm Al-Qura University, Makkah 21955, Saudi Arabia; hsamodi@uqu.edu.sa; 2 Clinical Pathology Department, National Liver Institute, Menoufia University, Menofia 32511, Egypt; hanan.bedier@liver.menofia.edu.eg; 3 Clinical Oncology and Nuclear Medicine Department, Faculty of Medicine, Menoufia University, Menofia 32511, Egypt; sozy.johar@med.menofia.edu.eg; 4 Medical Biochemistry and Molecular Biology Department, Faculty of Medicine, Ain Shams University, Cairo 11381, Egypt; dalia_wahab@med.asu.edu.eg; 5 Medical Biochemistry and Molecular Biology Department, Faculty of Medicine, Menoufia University, Menofia 32511, Egypt; eman.masoud@med.menofia.edu.eg; 6 Al Ryada University for Science and Technology (RST), ElMehwar ElMarkazy-2, Cairo—Alex Desert RD K92, Sadat City 16504, Egypt; mahmoud.nazih5698@pharm.aun.edu.eg; 7 Scientific Office, Egyptian Society of Pharmacogenomics and Personalized Medicine (ESPM), Cairo, Egypt; 8 Faculty of Pharmacy, Ahram Canadian University (ACU), 6th of October City, Giza 12566, Egypt; 9 Department of Clinical Pharmacy, Faculty of Pharmacy, Assiut University, Assiut 71526, Egypt; sahar.badr@pharm.aun.edu.eg; 10 Department of Clinical Pharmacy, Faculty of Pharmacy, Badr University in Cairo (BUC), Badr, Cairo 11828, Egypt; eman.said@buc.edu.eg; 11 Medical Microbiology and Immunology Department, Faculty of Medicine, Menoufia University, Menofia 32511, Egypt; dr_esraa_elmahdy87@yahoo.com; 12 Fellow at Pathology Department, National Liver Institute, Menoufia University, Menofia 32511, Egypt; asmaa.abdelmaksoud@osumc.edu; 13 Hematology Department, Faculty of Medicine, Menoufia University, Menofia 32511, Egypt; a.hassan14@med.menofia.edu.eg; 14 Clinical Biochemistry and Molecular Diagnostics Department, National Liver Institute, Menoufia University, Menofia 32511, Egypt

**Keywords:** *BAFF*, FCRL1, mRNA expression, DLBCL, B-cell non-Hodgkin lymphoma, R-CHOP therapy

## Abstract

This study investigated the diagnostic, prognostic, and therapeutic significance of Fc receptor-like 1 (*FCRL1*) and B-cell activating factor (*BAFF*) mRNA expression in Egyptian patients with diffuse large B-cell lymphoma (DLBCL) undergoing the standard R-CHOP regimen (rituximab, cyclophosphamide, doxorubicin, vincristine, and prednisone) using quantitative real-time PCR (RT-qPCR). The results demonstrated that *FCRL1* and *BAFF* mRNA expression were significantly elevated in DLBCL patients compared to healthy controls. A strong positive correlation existed between *BAFF* and *FCRL1* expression levels. Diagnostic performance assessed through combined ROC curve analysis revealed that *BAFF, FCRL1*, and lactate dehydrogenase (LDH) achieved perfect diagnostic accuracy (AUC = 1.0), demonstrating 100% sensitivity, specificity, and predictive values. Further prognostic analysis using COX regression identified elevated *FCRL1* expression as the most significant predictor of poor clinical outcomes. Kaplan–Meier survival analysis reinforced this finding, with high *FCRL1* expression showing significant associations with reduced overall survival (OS, *p* = 0.031) and progression-free survival (PFS, *p* = 0.038). The study underscores the potential utility of *BAFF* and *FCRL1* mRNA as diagnostic markers for DLBCL, with *FCRL1* emerging as a promising prognostic marker and potential therapeutic target enabling more tailored treatment approaches for DLBCL, the most common type of B-cell non-Hodgkin lymphoma, and patients receiving R-CHOP therapy.

## 1. Introduction

B-cell non-Hodgkin lymphoma (B-NHL) is a significant global health challenge due to its rising incidence and associated mortality. Recent epidemiological data reveal that approximately 450,000 new cases are diagnosed annually all around the world, with nearly 240,000 recorded deaths [1]. Diffuse large B-cell lymphoma (DLBCL), the most common subtype of B-cell non-Hodgkin lymphoma, accounts for a significant proportion of cases among the seven major types of B-cell lymphomas, which include follicular lymphoma, chronic lymphocytic leukemia, mantle cell lymphoma, marginal zone lymphoma, Burkitt lymphoma, Waldenstrom macroglobulinemia, and hairy cell leukemia. Diffuse large B-cell lymphoma (DLBCL) is the most prevalent and aggressive of the B-NHL subtypes, making up a significant percentage of cases. DLBCL is described as marked clinical and biological variability, complicating its management and prognosis [2].

Despite advances in treatment, a significant subset of patients continues to experience poor outcomes, underscoring the urgent need for improved therapeutic strategies and prognostic tools [3].

The advent of rituximab in combination with cyclophosphamide, doxorubicin, vincristine, and prednisone (R-CHOP) has transformed the treatment landscape of DLBCL, achieving remarkable survival improvements; however, 40% of patients eventually experience relapses or develop intractable [4,5]. Relapses of this kind are frequently linked to poor prognosis and few available treatment choices. It has been suggested that molecular heterogeneity within DLBCL contributes significantly to these suboptimal outcomes, highlighting the need for biomarkers capable of predicting responses to treatment and enabling personalized therapy [6].

Current knowledge points to the dysregulation of several critical signaling pathways in B-NHL pathogenesis and resistance to R-CHOP treatment. According to earlier research, the Janus kinase (JAK)/signal transducer and activator of transcription 3 (STAT3) pathways, abnormal signaling of the B-cell receptor (BCR), nuclear factor kappa light chain enhancer of activated B-cells (NF-κB), and phosphatidylinositol 3-kinase (PI3K)/Akt are possible molecular mechanisms of R-CHOP resistance and B-NHL diversity [7].

Therefore, it is crucial to find the best biomarkers to identify the remaining patients who were not cured with R-CHOP treatment and explore new therapeutic targets to decrease the treatment resistance or failure [2]. The International Prognostic Index (IPI) is the major clinical risk assessment instrument used to determine variances in non-Hodgkin lymphoma patients’ responses to various treatment modalities. It is based on lactate dehydrogenase, performance status (PS), number of extranidal sites, age, and Ann Arbor stage. Although it is an effective tool in predicting survival, it can not help tailoring personalized therapies and targets [8].

The search for novel molecular markers has gained momentum in this context, with Fc receptor-like 1 (*FCRL1*) and B-cell activating factor (*BAFF*) emerging as promising candidates. Studies performed on molecular analysis have lately indicated that the *(FCRL1*) gene is involved in B-cell-derived hematological malignancies [9,10,11,12,13,14]. Additionally, FCRL1 receptors are lymphocyte receptors that are valuable targets for different B-cell-related conditions categorization or immunotherapy [15,16,17,18,19].

FCRL1 comprises two immunoreceptor tyrosine-based activation motifs (ITAM)-like components located in its intracellular tail overexpressed favorably in B cells [12,18]. According to a paper, FCRL1 may activate B-cells when its overexpression positively correlates with the up-regulation of its co-receptors, such as CD80 and CD69, in B cell lines [20]. Furthermore, FCRL1 has many vital roles in immunity and carcinogenesis by affecting apoptosis, epithelial-mesenchymal transition (EMT), and DNA repair [21]. Notably, it was reported that FCRL1 supports humoral responses and regulates B cell signaling. Many advancements to identify FCRL1 regulatory characteristics and the evidence of its over-expression in B cell lymphoma reflect essential functions of FCRL1 in B cell development [22].

Similarly, BAFF, sometimes called B-lymphocyte stimulator (BLyS), exists in two forms, either on the cell surface or soluble in the serum. It belongs to the family of tumor necrosis factors (TNFs) [23] and encourages B-lymphocyte survival and maturation as it may stimulate B cells and promote their growth, contributing to the pathophysiology of NHL. BAFF-receptor (BAFF-R) is the key receptor critical for B-cell maturation. The BAFF/ BAFF-R pathway plays an important role in the survival and growth of mature B-cells. Furthermore, *BAFF* overexpression is indicated in many autoimmune and neoplastic disease developments like Sjögren’s syndrome (SS) and systemic lupus erythematosus (SLE) [24].

Unusual expression of *FCRL1* and *BAFF* was stated in diffuse large B cell lymphoma (DLBCL) cell lines and Burkitt lymphoma (BL) [12,13,17] as well as follicular lymphoma (FL), mantle cell lymphoma (MCL), chronic lymphocytic leukemia (CLL), hairy cell lymphoma (HCL), and multiple myeloma (MM) [12,13]. These findings suggest that *FCRL1* and *BAFF* play a significant part in the etiology or development of B-cell cancers. However, these findings had implications for earlier research [25].

Preliminary research indicates that *FCRL1* and *BAFF* may act synergistically in B-cell malignancies, as their signaling pathways overlap significantly in promoting tumor survival and resistance mechanisms. Research studies have demonstrated a strong association between the expression levels of these markers in B-NHL, suggesting their combined utility in diagnosis and prognosis [22]. However, while their individual roles have been partially explored, the prognostic significance of their co-expression, particularly in patients receiving R-CHOP treatment, remains under investigation.

Therefore, the current study aimed to determine the diagnostic and prognostic functions of *FCRL1* and *BAFF* in Egyptian patients with diffuse large B-cell lymphoma (DLBCL), the most prevalent subtype of B-cell non-Hodgkin lymphoma. (B-NHL), receiving R-CHOP treatment, revealing new targets for individualized treatment strategies, and offering insights into the molecular causes of drug resistance.

## 2. Results

### 2.1. Demographic Data and Characteristics

Our study included 100 participants, 40 of whom served as a control group, and 60 patients diagnosed with DLBCL were included in this study. Neither gender distribution nor mean age showed statistically significant differences between non-Hodgkin lymphoma patients and control groups (*p* = 0.307, 0.19, respectively) (Table 1).

### 2.2. Tumor Characteristics

The Lugano staging system was employed to categorize the extent of the lymphoma. Most cases were observed in Stage IA (31.7%), followed by Stage IVA (21.7%). Extra-nodal involvement was detected in 25% of the cases, with liver and lung being the most common sites (Table 2).

Performance status scores were 0 in 53.3%, 1 in 38.3%, and 2 in 8.3% of cases. This study reported that 46.7% of the patients had low-category IPI, followed by low intermediate and high intermediate (35% and 16.7%, respectively) and high intermediate (1.7%).

### 2.3. BAFF and FCRL 1 mRNA Were Overexpressed in DLBCL Patients

**Figure 1:**
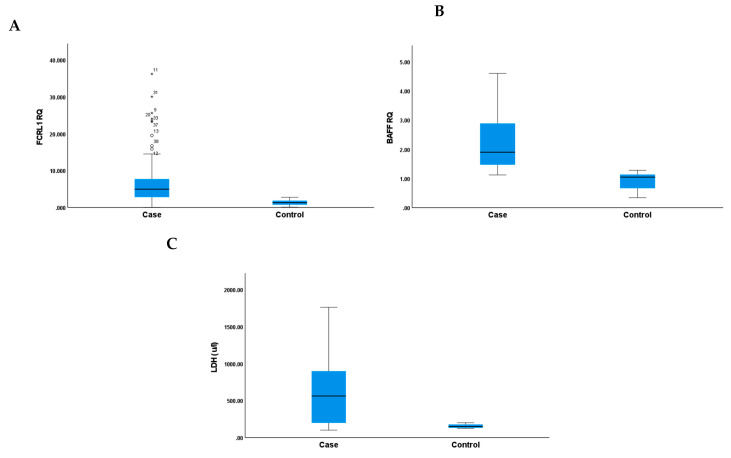
(**A**) Fc receptor-like 1(*FCRL1*) RQ, (**B**) B-cell activating factor (*BAFF*) RQ, and (**C**) lactate dehydrogenase (LDH) level distribution among cases and control groups. Mann–Whitney U test compares *FCRL 1, BAFF*, and LDH expressions between groups.

To investigate the expression levels of *BAFF* and *FCRL* 1 mRNA, qRT-PCR was performed in PMBCs from patients and healthy controls. FCRL1 relative expression levels were significantly greater in patients than normal samples (median (IQR) = 4.96 (5.03)) and (median (IQR) = 1.3 (1.19), respectively (*p* < 0.001; Figure 1A). In addition, *BAFF* RQs were greater in patients than controls (median (IQR)= 1.9 (1.47)) and (median (IQR) = 1.045 (0.49), respectively (*p* < 0.001; Figure 1B). Similarly, LDH levels were markedly elevated in the DLBCL group (median (IQR) = 561.5 (701)) than healthy controls (median (IQR) = 150 (47)) (*p* < 0.001 Figure 1C).

### 2.4. BAFF and FCRL 1 mRNA Diagnostic Accuracy in DLBCL Patients

**Figure 2:**
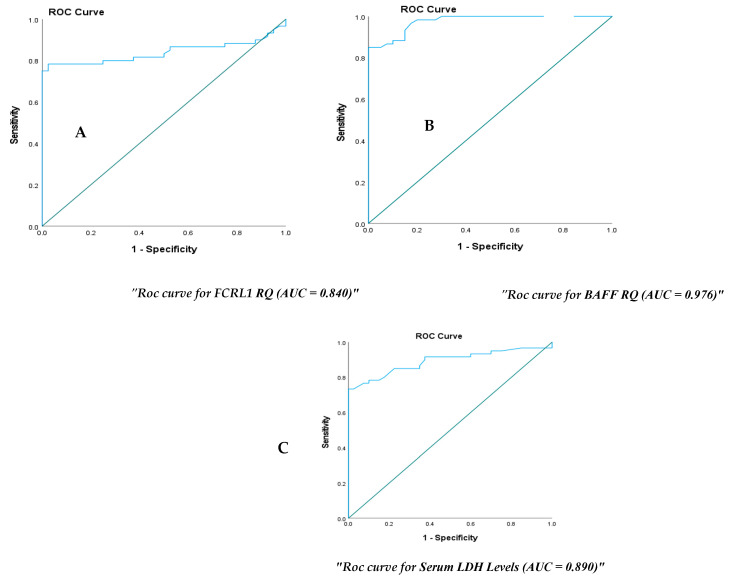
ROC curve analysis for fold change of Fc receptor-like 1 (*FCRL 1*), B-cell activating factor (*BAF*F) RQ, and LDH levels to discriminate DLBCL patients from healthy controls; (**A**): ROC curve for diagnosis of DLBC using fold change of FCRL1, AUC = 0.840, cut off value≥2.3; (**B**): ROC curve for diagnosis of DLBCL using fold change *BAFF* mRNA, AUC = 0.976, cut off value ≥1.16; (**C**): ROC curve for diagnosis of DLBCL using LDH serum level, AUC = 0.890, cut off value ≥179 (U/L).

As shown in Table 3, BAFF demonstrated high diagnostic accuracy, achieving an AUC of 0.976 (95% CI: 0.95–0.99) at a cutoff point of 1.16, with sensitivity and specificity of 93.3% and 85%, respectively (Figure 2A). While *FCRL 1* had a cutoff point of 2.32 and exhibited a fair diagnostic accuracy with an AUC of 0.840 (95% CI: 0.756–0.924), with a sensitivity of 78% and a specificity of 98% (Figure 2B). Moreover, LDH yielded an AUC of 0.89 (95% CI: 0.82–0.95) at a cutoff point of 179 u/L, with a sensitivity of 85% and a specificity of 77% (Figure 2C).

The AUC for the studied biomarkers, sensitivity, specificity, and positive and/or negative predictive values were all 100% on combination analysis.

### 2.5. Correlation Between BAFF and FCRL 1 Expression Levels in DLBCL Patients

**Figure 3:**
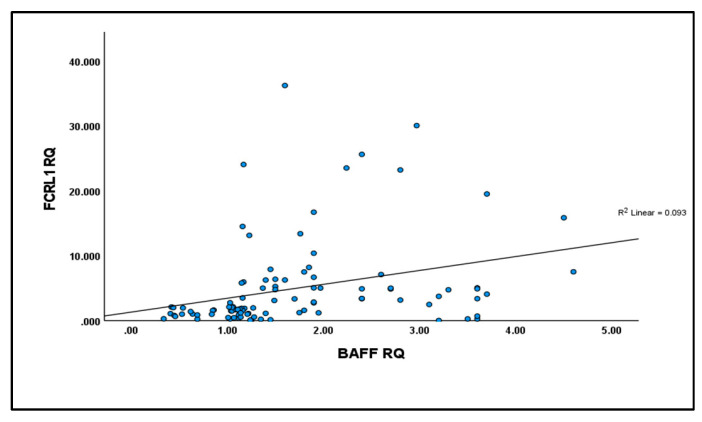
*FCRL*1mRNA has a significant direct correlation with *BAFF* expression in PMBCs with *p* = 0.0.

As seen in Figure 3, the results showed a significant direct correlation between the expression levels of *BAFF* and *FCRL* 1 (r = 0.5, *p* < 0.001).

### 2.6. Association Between BAFF Expression, FCRL1 Expression, and LDH with Different Clinical Criteria

Notably, LDH levels exhibit significant associations with the modified Lugano staging and the International Prognostic Index (IPI). Neither *FCRL* 1 nor *BAF*F expression exhibited significant correlations with any clinical parameters such as modified Lugano Staging, extra-nodal site involvement, PS, or IPI scores (*p* > 0.05) (Table 4).

### 2.7. Survival Analysis for BAFF, FCRL 1 Overexpression, and LDH Levels

To evaluate the prognostic significance of these biomarkers, patients were stratified using median expression values as cutoff points. For *FCRL*1, the median expression level of 4.95 was used to categorize patients into high-expression (n = 30) and low-expression (n = 30) groups. Similarly, patients were divided at the median value of 1.90 for BAFF expression into high-expression (n = 30) and low-expression (n = 30) groups. For serum LDH levels, a median cutoff point at 561.5 U/L was used to stratify patients into high-level (n = 30) and low-level (n = 30) groups. Survival analysis was conducted using the Kaplan–Meier methodology with log-rank tests to compare survival distributions.

The survival outcomes and treatment responses demonstrated significant associations with *FCRL1* expression levels. The estimated 3-year OS rate was 75%, with a mean survival time of 31.5 months (95% CI: 28.9–34.1). Notably, high *FCRL1* expression significantly correlated with lower OS (*p* = 0.031), with a hazard ratio of 1.966 (95% CI: 1.041–3.713) (Table 5). Treatment response rates showed marked differences between *FCRL1* expression groups, with low-expression patients achieving a significantly higher complete response rate (86.7%) compared to high-expression patients (63.3%) (*p* = 0.031).

**Figure 4:**
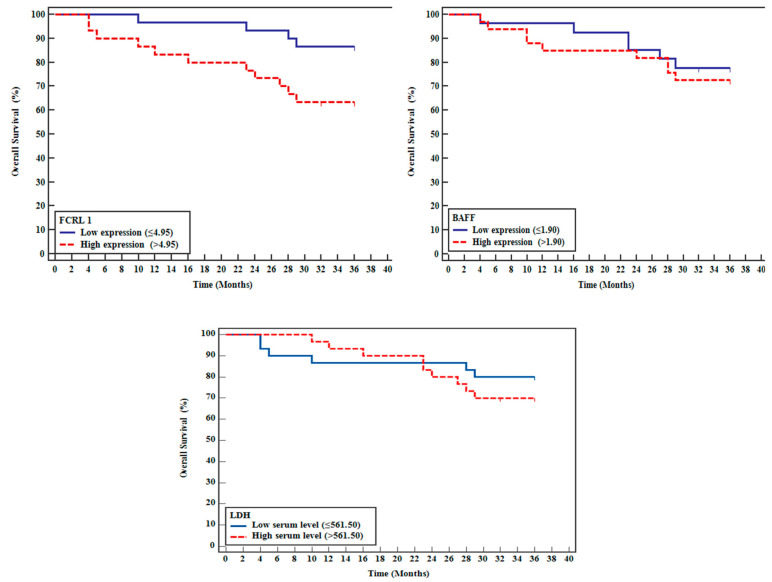
Kaplan–Meier survival curve for overall survival with *FCRL 1, BAFF*, and LDH.

**Figure 5:**
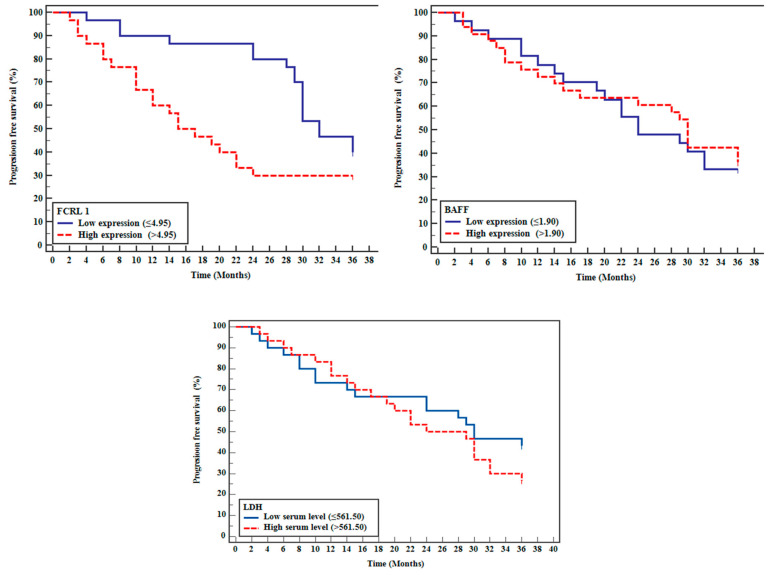
Kaplan–Meier curve for progression-free survival with *FCRL1, BAFF*, and LDH.

These findings highlight the critical impact of *FCRL1* on survival outcomes and therapeutic efficacy, suggesting its potential as a biomarker for tailoring treatment strategies in DLBCL, the most common type of B-NHL (Figure 4 and Figure 5 and Table 6 and Table 7).

Progression-free survival (PFS) analysis revealed an estimated 3-year PFS rate of 34.5%, with a mean of 24.27 months. Patients with high *FCRL1* expression exhibited significantly shorter PFS than those with lower expression levels (*p* = 0.038). Competing risk analysis demonstrated that *FCRL1* retained its prognostic significance for PFS after adjusting for death as a competing event, further reinforcing its relevance in outcome prediction (Figure 5 and Table 7).

### 2.8. COX Regression Analysis for Different Factors Affecting Poor Outcomes

Notably, *FCRL 1* overexpression is significantly associated with poor outcomes (hazard ratio = 1.966, *p* = 0.037), revealing that its upregulation can be used as an independent prognostic factor for predicting worse outcomes in DLBCL, the most common type of B-NHL patients (Table 7).

### 2.9. Subgroup Analysis Results

Analysis of patient subgroups revealed the consistent prognostic significance of *FCRL1* expression across different clinical parameters. As shown in Table 4, analysis of clinical factors demonstrated that while LDH levels exhibited significant associations with modified Lugano staging and IPI, *FCRL1* expression maintained its independent prognostic value (HR = 1.966, *p* = 0.037) regardless of other clinical parameters. This finding was further supported by Kaplan–Meier survival analysis (Figure 4 and Table 6), which demonstrated significantly worse overall survival in patients with high *FCRL1* expression (log-rank test Chi-square = 4.650, *p* = 0.03).

The prognostic impact of *FCRL1* was further validated in progression-free survival analysis (Figure 5 and Table 7), where the log-rank test remained significant (chi-square = 4.324, *p* = 0.038), confirming *FCRL1’s* role as an independent prognostic marker in DLBCL patients.

## 3. Discussion

B-NHL is a malignancy with different clinical consequences [26]. Many studies have been carried out to explore these B-cell malignancy etiologies and mechanisms to improve their prognosis and treatments. Most patients have good responses to established therapies. However, there is a need for treatments suitable for aggressive, resistant, and relapsed kinds of B-cell lymphomas [4]. Moreover, further studies are required to explore new biomarkers for diagnosis and prognosis for B-NHL cancer.

The current research aimed to investigate whether FCRL1 or BAFF is a suitable target for diagnosing, prognosticating, and improving therapeutic outcomes in Egyptian DLBCL, the most common type of B-NHL, patients treated with R-CHOP.

This study was carried out on 60 non-Hodgkin lymphoma patients and 40 healthy controls coordinated regarding sex and age. DLBCL patients were categorized according to the modified Lugano staging system that distinguishes the extent of lymphoma in these patients. Most cases were noted in Stage IA (31.7%), followed by Stage IVA (21.7%). Moreover, extra-nodal involvement was found in 25% of the cases, with liver and lung being the most common sites. Lugano classification was issued for the first time in 2014 to inform the foundation for anatomic staging and assessment of malignant lymphoma before and after treatment. That staging system was accepted by the eighth edition of the Cancer Staging Manual of the American Joint Committee on Cancer [27].

Our study reveals three principal findings regarding FCRL1 and BAFF expression in DLBCL of B-NHL. First, compared to controls, we demonstrated significant overexpression of both *FCRL1* and *BAFF* mRNA in DLBCL patients. Second, we identified a novel positive correlation between FCRL1 and BAFF expression (r = 0.5, *p* < 0.001), suggesting potential co-regulation. Third, high *FCRL1* expression emerged as an independent prognostic factor (HR = 1.908, 95% CI: 1.007-3.613, *p* = 0.047), consistent with recent findings in other B-cell malignancies [21].

This study showed that *FCRL1* and *BAFF* mRNA expression levels were significantly higher in lymphoma patients than in controls. Yousefi and colleagues examined the FCRL1 expression in B-NHL patients by quantitative PCR (qPCR). They also added that *FCRL1* expression was higher in patients with hairy cell leukemia, diffuse large B-cell lymphoma, and Burkitt lymphoma cases than controls [23]. Another interesting study by Yousefi et al. revealed that *FCRL1* knockdown considerably reduced cell proliferation and raised apoptosis in the Burkitt lymphoma (BL) cell lines. These effects were due to a significant decrease in pro-survival Bcl-2, phosphorylated-p65 NF-κB activity, and the expressions of PI3K/p-AKT. Meanwhile, cells boosted the pro-apoptotic Bid and Bax expression levels in the group of treated BL cells [21].

Much evidence of a potential link between *FCRL1* and B-cell tumors has been detected [28]. A probe of the “lymphochip” and related microarray studies by Alizadeh and colleagues found differential overexpression of FCRL1 mRNA in many aggressive B cell lymphoproliferative malignancies [29,30]. However, *FCRL1* expression is lower in acute lymphoblastic leukemia (ALL) and multiple myeloma, regardless of its distribution during normal B cell development [17].

The B-cell activating factor (BAFF) is an important player in the evolution of B-cells and their survival [20]. *BAFF* overexpression has been identified in different malignancies and is related to severity and treatment outcome [31]. The expression of BAFF has been principally investigated in MM and CLL, but its influence on other malignancies is still progressing. BAFF and its receptors up-regulating pro-survival (Bcl-2, Bcl-xL) as well as growth-promoting (c-Myc) proteins while down-regulating pro-apoptotic Bax in both Hodgkin lymphoma cells and acute lymphoblastic leukemia. Moreover, it also lessens the sensitivity of cancer cells to chemotherapeutics [32,33].

Moreover, LDH levels were markedly higher in the DLBCL group than in healthy controls. Similarly, a study by Mohammed and Hamodat revealed a significant elevation in LDH levels for NHL patients compared to controls. Furthermore, they found elevations of LDH levels in untreated NHL cases compared to treated NHL cases. A non-significant variance was reported between treated NHL cases and the control group. These results imply that LDH may be utilized as an indicator for patient diagnosis and therapy monitoring [34].

These results parallel former molecular analysis research showing that the overexpression of *FCRL1* and *BAF*F is implicated in encouraging the proliferation and progression of B-NHL via various signaling pathways, suggesting them as promising immunotherapeutic targets for DBCL [35].

In the same context, Du et al. investigated targeting hFCRL1 using a 38-kDa fragment of Pseudomonas exotoxin A (PE38) amalgamated to single-chain variable fragments (scFv) cloned from mAbs to create recombinant immunotoxins. The cytotoxicity of these immunotoxins was related to expression levels of hFCRL1, and the binding affinities were stable over time [36]. According to the researchers, these features were like the cytotoxic state of other immunotoxins studied in many clinical trials, which supports the idea that hFCRL1 could be a target for immunotherapy. Targeting hFCRL1 will more likely play a breakthrough in this area, particularly since other frequently targeted B cell-restricted antigens may gradually decrease or disappear from patients’ cell surfaces [37].

ROC curve analysis was performed to detect the diagnostic accuracy of *BAFF* and *FCRL1* mRNA relative expression levels and LDH serum levels. BAFF mRNA established a diagnostic accuracy with an AUC of 0.976 (95% CI: 0.95–0.99) at a cutoff point of 1.16, with sensitivity and specificity of 93.3% and 85%, respectively (Figure 2A). FCRL 1 had a cutoff point of 2.32 and showed a fair diagnostic accuracy with an area under the curve (AUC) of 0.840 (95% CI: 0.756–0.924), with a sensitivity of 78% and a specificity of 98% (Figure 2B). Moreover, LDH serum levels have an AUC of 0.0.89 (95% CI: 0.82–0.95) at a cutoff point of 179 u/L, with a sensitivity of 85% and a specificity of 77% (Figure 2C).

The diagnostic accuracy of combined *FCRL1/BAFF* testing (100% sensitivity and specificity) suggests potential utility in monitoring disease progression and treatment response. This is particularly relevant given recent findings showing variable outcomes with different R-CHOP regimens [38], highlighting the need for better patient stratification. Intriguingly, the current results showed a positive association between *BAFF* and *FCRL1* expression levels in DLBCL patients. These results indicated the essential roles of BAFF and FCRL 1 mRNA in carcinogenic effects in B-cell non-Hodgkin lymphoma. The observed correlation between *FCRL1* and *BAF*F expression suggests a previously unrecognized molecular axis in B-NHL pathogenesis. This interaction may involve convergent signaling pathways, particularly through NF-κB and PI3K/AKT networks, critical for B-cell survival and proliferation. Understanding this relationship could reveal new therapeutic vulnerabilities in B-NHL.

Our study presents new insights into the molecular interplay between *FCRL1* and BAFF in DLBCL pathogenesis. For the first time, we demonstrated their individual overexpression and their coordinated expression patterns, suggesting a potentially synergistic role in disease progression.

These new insights into the molecular underpinnings of DBCL biology suggest that targeting the *FCRL1/BAFF* axis may represent a novel therapeutic strategy of personalized approaches to lymphoma treatment, particularly for high-risk patients who exhibit poor responses to conventional R-CHOP therapy.

Moreover, the current study analyzed the relationship between the two studied biomarkers’ expression levels, LDH serum levels, and different clinic pathological parameters. There were non-significant correlations in *FCRL1* or *BAFF* mRNA expression regarding age, modified Lugano Staging, extra-nodal site involvement, performance status, or IPI scores. Notably, LDH correlated positively with modified Lugano staging and IPI.

On the contrary, Yousefi and Eskandari’s study found a positive association between FCRL1 relative expression levels and age in B-NHL patients [23]. Additionally, Yousefi et al., reported a positive correlation between expression level of FCRL1 and many clinicopathological criteria of large B-cell lymphoma patients, such as “tumor size, stage of disease, PS at diagnosis, and IPI scores” [21].

The current study revealed that OS time was lower in patients with relatively high expression levels of *FCRL 1 or BAFF* (above median) than low relative expression levels. Additionally, Kaplan–Meier curve analysis for PFS time with *FCRL1* and *BAFF* revealed survival was lower in patients with high expressions of FCRL1 BAFF. Moreover, COX regression analysis for different factors affecting poor outcomes revealed that FCRL 1 overexpression is significantly associated with poor outcomes (Hazard ratio = 1.966, *p* = 0.037), revealing that *FCRL 1* and/or *BAFF* therapeutic targeting may overcome such worse outcomes in DLBCL patients.

Seok Jin Kim et al. reported that BAFF might be related to the resistance of DLBCL to R-CHOP as their results showed that three patients showed a full response in the high BAFF group, compared to 21 in the low BAFF group. Also, eleven of the sixteen recurrence patients were in the high BAFF group. Additionally, relapsed patients had higher BAFF receptor expression than the non-relapse group. Thus, the response of DLBCL to R-CHOP may be linked to baseline serum BAFF [39]. Similarly, Schmidt et al. suggested that one definite mechanism of resistance to rituximab refers to decreased sensitivity to rituximab-induced antibody-dependent cell-mediated cytotoxicity caused by BAFF l production of natural killer cells [40,41].

Besides, the *FCRL1* expression prognostic significance has important risk stratification and treatment selection implications. Our findings suggest that *FCRL*1 expression could help determine high-risk B-NHL patients who might benefit from treatment intensification. Recent meta-analyses have shown that dose-intensive approaches can improve outcomes in specific B- NHL, especially DLBCL subgroups (primary mediastinal B-cell lymphoma) [42].

Kaplan–Meier curve analysis was undertaken for OS and PFSl times with *FCRL1*, *BAFF*, and LDH levels. The estimated OS from the time of diagnosis was 75% at 3 years with a mean of 31.5 months, which is nearly like that found by Musimar et al. [25] and a Swedish group and others [43], possibly due to the short follow up period 3 years instead of 5 years. Meanwhile, the estimated PFS rate at 3 years was 34.5%, with a mean of 24.27 months. Additionally, the log-rank test was statistically significant for FCRL 1 only. Furthermore, DLBCL patients were categorized into high and low expression groups regarding the median expression levels of *BAFF* and *FCRL 1* and the median serum level of LDH, which showed the overall survival time. PFS were lower in DLBCL patients with high relative expression levels of *FCRL 1* or *BAFF* than low relative expression levels. Moreover, survival time and PFS for DLBCL patients with high serum levels of LDH were less than those with low serum levels of LDH.

These results agree with Smith et al., who suggested that the standard treatment, including rituximab, might better modify survival despite limited healthcare resources [44]. Meanwhile, miserable compliance and management pause in low resources (Africa) are usually related to the disease’s recurrence and/or progression and survival [45]. So, there is a demand for improved medications for DLBCL in the relapsed or refractory status, e.g., larger chemotherapy doses with replacement of bone marrow and future therapies like antibody-drug combinations [25].

Furthermore, we assessed the influence of IPI, age, PS, *BAFF*, and *FCRL1* mRNA relative expression levels and serum LDH levels on survival to be accepted as prognostic factors in our study. Poor survival was significantly associated with high *FCRL1* mRNA relative expression levels. These findings can allow the early identification of poor-risk patients suitable for alternate treatment strategies [46].

Opposing our results, Martínez et al. stated that plasma levels of BAFF were univariately related to overall survival and correlated with progression. They also found that OS was significantly lower in B-NHL patients with high BAFF levels in non-Hodgkin lymphoma, so it could provide a potential prognostic biomarker in those patients [47].

However, Musimar et al. reported that performance status, not IPI or age, was significantly related to survival in DLBCL patients. They explained the absence of a significant outcome of IPI on OS by the small sample size [25]. Additionally, other studies reported that PS was more valuable in expecting outcomes than age [44,48]. Likewise, as reported by the GELA study, IPI was not a momentous element in predicting the OS rate at 2 years for DLBCL patients receiving R-CHOP [49,50]. This promotes a finding for improving new prognostic modalities [25]. In addition, univariate and multivariate COX regression analysis of the variables affecting poor outcomes revealed that the most predictor factor was the high expression of FCRL 1 mRNA.

Recent developments in targeting hFCRL1, such as Du et al.’s work with recombinant immunotoxins using PE38 fused to single-chain variable fragments, show promising therapeutic potential [36]. The stable binding affinities and correlation with h*FCRL1* expression levels suggest potential clinical applications [36]. Through meta-analyses, Cook et al. demonstrated that dose-intensive approaches could improve outcomes in specific B-NHL subgroups [42]; therefore, our study suggests that FCRL1 expression might help identify suitable candidates for treatment intensification.

Identifying *FCRL1* as an independent prognostic factor in molecular mechanisms has important implications for personalized medicine approaches in DLBCL. High *FCRL1* expression might identify patients who could benefit from novel targeted therapies or more intensive treatment regimens. The correlation with BAFF expression suggests that dual targeting of these pathways might be more effective than single-agent approaches.

Our 100% diagnostic accuracy finding with combined *FCRL1/BAFF* testing represents a significant advance in DLBCL diagnostics. The independent prognostic value of *FCRL1* expression, particularly its association with inferior survival in R-CHOP-treated patients, suggests its potential utility in identifying high-risk patients who might benefit from alternative therapeutic strategies.

The findings of this study open several promising avenues for upcoming research to advance the clinical utility of *FCRL1* and *BAFF* in non-Hodgkin lymphoma (NHL), translating molecular insights into actionable strategies for improving diagnostic accuracy, prognostic precision, and therapeutic outcomes in DLBCL patients, especially. By addressing key gaps in understanding and clinical practice, this research has the potential to shape a more personalized and effective approach to lymphoma management.

### Limitation of This Study

While our study provides valuable insights into the prognostic significance of *FCRL1* and *BAFF* expression in lymphoma patients, several limitations warrant consideration. First, the molecular heterogeneity between different NHL subtypes suggests that our findings, primarily derived from DLBCL patients, may have varying applicability across other lymphoma classifications. BAFF expression levels differ significantly among lymphoma subtypes, with notably different patterns observed in B-CLL and follicular lymphoma compared to DLBCL, MCL, and marginal zone lymphoma. Similarly, FCRL1 expression demonstrates subtype-specific variations that merit further investigation.

Second, while the sample size (n = 60) provided sufficient statistical power for our primary analyses, larger multicenter cohorts would be valuable for validating these findings and exploring potential subset-specific effects. This is particularly relevant for stratified analyses examining the interaction between biomarker expression and specific clinical characteristics.

Third, the follow-up period of 36 months, though adequate for initial survival analyses, may not capture very late relapses or progression events. Extended follow-up would be valuable for assessing long-term prognostic implications, particularly given the heterogeneous natural history of lymphomas.

Fourth, our analysis focused on mRNA expression levels in peripheral blood mononuclear cells. Although this approach offers practical advantages for clinical implementation, complementary analyses of protein expression and tissue-specific patterns could provide additional insights into the biological significance of these markers.

Finally, while our findings suggest potential therapeutic implications, particularly regarding R-CHOP response prediction, prospective studies are needed to validate the clinical utility of *FCRL1* and *BAF*F expression-based patient stratification in treatment decision-making.

## 4. Materials and Methods

### 4.1. Study Patients

The sample size for this prospective cohort study was calculated using G*Power 3.1 software. Based on prior studies of *FCRL1* expression in lymphoma, an expected effect size of 0.6 was used, with α = 0.05 and power (1-β) = 0.80, to detect a 1.5-fold difference in expression levels between groups [23]. The calculation indicated a minimum requirement of 52 patients; however, we enrolled 60 patients to account for potential dropouts and ensure the robustness of the study.

This study was conducted on sixty Egyptian patients with histopathological confirmation of diffuse large B-cell lymphoma (DLBCL). All the included patients were ≥18 years old and recruited from Clinical Oncology, Hematology Unit of Internal Medicine, and Clinical Pathology departments of Menoufia University hospitals, in addition to 40 age and sex well-matched healthy subjects served as a control group.

Before their study enrollment, each subject provided written informed consent. The Menoufia University Faculty of Medicine’s ethics committee authorized our study’s protocol, and its Institutional Research Board number was (2/2023 ONCO24-1).

For diagnosis, an incisional and/or excisional biopsy was obtained from the patients involved in this study. Immunohistochemistry was performed for CD20 positivity, which was a requisite for patients treated with rituximab. Computed tomography (CT) or positron emission tomography (PET-CT) imaging, bone marrow aspirate, and trephine biopsy were carried out for staging.

End-stage renal disease, liver cell failure, and heart failure patients were excluded. Also, patients who proved to be HIV positive or previously received chemo or radiotherapy were barred. All patient groups were subjected to a full history taking, clinical examination, and laboratory investigations. Performance status (PS) and the international prognostic index (IPI) were assessed, and the patient group was staged according to the Ann Arbor system (modified Lugano staging system) [24].

All patients received the treatment regimen that consisted of rituximab combined with cyclophosphamide, vincristine, and doxorubicin, in addition to prednisone (R+CHOP). The recommended protocol for Stage I/II (non-bulky) DBCL consists of R+CHOP for 3–4 cycles followed by involved field radiation therapy (IFRT). However, advanced stages such as bulky stage II DBCL or (stage III-IV) receive R+CHOP every 21 days for 6 cycles, with or without IFRT for bulky places. Intrathecal (IT) chemotherapy can be used prophylactically in selected cases. Rituximab was handled at the dose of 375 mg/m^2^ on day 1, and CHOP chemotherapy was in combination with rituximab for 6 cycles.

All patients were followed for at least 36 months from diagnosis and underwent clinical examination every cycle for signs of treatment toxicity and clinical response.

Complete remission (CR) was characterized as the disappearance of all lesions for 4 weeks. Partial remission was characterized as a decrease in lymph node mass by at least 50%, whereas progressive disease was characterized by the presence of new lesions or increased lymph node size. Patients with no CR, partial remission, or progressive disease lesions were classified as having stable diseases.

Progression-free survival (PFS) was calculated as the duration of time from the start of treatment until disease progression in the presence of relapse developed new nodal/extra-nodal sites or increase in size and/or the number of involved lymph nodes previously. Overall survival (OS) was dictated as the time from chemotherapy imitation until the date of the patient’s last follow-up visit or death [25].

### 4.2. Blood Sampling

Five milliliters (mL) of peripheral blood were collected from the participants and divided into two fractions: 2 mL placed into a plain vacutainer tube for serum separation and subsequent lactate dehydrogenase (LDH) measurement using particle-enhanced immunoturbidimetric assay utilizing Cobas e 601 Auto analyzers (Roche Diagnostics GmbH, Mannheim, Germany) and the remaining 3 mL was placed into a tube holding ethylene diamine tetra acetic acid (EDTA) for peripheral blood mononuclear cells (PBMCs) isolation and subsequent gene expression analysis.

### 4.3. Peripheral Blood Mononuclear Cells (PBMCs) Isolation

PBMCs were separated by Ficoll density gradient centrifugation (Amersham-Biosciences, Uppsala, Sweden). Then, PBMCs were extracted, three phosphate-buffered saline (PBS) washes were performed, and cells were kept at −80 °C for subsequent investigation. Expression levels of *FCRL1* and *BAFF* mRNAs in PBMCs isolated from patients and controls were relatively measured by the quantitative reverse transcriptase polymerase chain reaction (qRT-PCR) technique.

### 4.4. Extraction of Total RNA from PBMCs and Reverse Transcription

RNA was extracted from PBMCs using RNeasy plus Universal Kit (QIAGEN GmbH, Hilden, Germany; Cat. No. 73404). RNA concentration and purity were checked using a NanoDrop 1000 spectrophotometer (Thermo Fisher Scientific Inc., Waltham, MA, USA). by measuring absorbance ratios at 260/280 nm. cDNA was yielded by the reverse transcription of the RNA utilizing SensiFAST cDNA Synthesis Kit (Bioline Reagents Ltd., Quantiscript Reverse Transcriptase London, UK; Cat. No. BIO-65053). A final volume of 20 microlitres (μL) was prepared: 1 μL of reverse transcriptase enzyme, 4 μL of the Buffer, 10 μL of RNA template, and 5 μL of nuclease-free water. The reverse transcription was performed using a 2720 thermocycler (Applied Biosystems, Thermo Fisher Scientific, Singapore). with the following cycling conditions: initial incubation at 42 °C for 10 min, followed by heat inactivation at 95 °C for 5 min, and final cooling at 4 °C for 5 min. The produced cDNA was preserved at −20 °C until qPCR was applied.

### 4.5. Quantitative Expression of FCRL1 and BAFF mRNA Using Real-Time Quantitative PCR (RT-qPCR)

RT-qPCR was utilized for a relative measure of *FCRL1* and *BAFF* mRNA expressions in the PBMCs in the studied participants via SensiFAST SYBR Lo-ROX Kit (Columbia, SC, USA Bioline Reagents Cat. No. BIO-94005). Ten μL of SYBR green Master Mix, one μL of Nuclease free water, six μL of template cDNA, and 1.5 μL of each primer (sense and antisense) were mixed, producing 20 μL final volume. The following oligonucleotide primers were: 5’-CCTACCTACACTCACCTAC-3′ (sense), 5′-TCTGCTGCTACTGATTCC-3′ (antisense) for *FCRL1*; 5′-CACGCCTTACTTCTTGCC-3′ and 5′-CTTGGAGGATCGGACAG-3′ for *BAFF* (antisense). Lastly, 5′-TCAAGGCTGAGAACGGGAAG-3′ (sense), 5′-GTGAAGACGCCAGTGGACT-3′ (antisense) for glyceraldehyde 3-phosphate dehydrogenase (*GAPDH*) as a housekeeping internal control. The amplification steps were carried out in this way: an initial activation time at 95 °C for 5 min, then by 45 cycles at 95 °C for 20 s, 60 °C for 30 s, and 72 °C for 1 min. Finally, the extension phase was performed at 72 °C for 10 min by RT-PCR 7500 ABI PRISM (Applied Biosystems, Thermo Fisher Scientific, Waltham, MA, USA). v.2.0.1. Relative quantification (RQ) was calculated using the 2^−ΔΔCT^ method according to [51] utilizing *GAPDH* as an internal housekeeping gene.

### 4.6. Statistical Analysis

Data were analyzed using the IBM SPSS Statistics for Windows version 27 (IBM Corp., Armonk, NY, USA). Frequencies and percentages were provided for categorical data, while mean and standard deviation (SD) or medians and interquartile ranges (IQR) were shown for continuous parameters. Pearson’s Chi-square test was used to determine the associations between the categorical variables. Meanwhile, the independent sample T-test and Mann–Whitney U test were employed to measure continuous variables. The diagnostic accuracy of FCRL 1, BAFF, and LDH was assessed by the receiver operating characteristic (ROC), determining the area under the curve (AUC), sensitivity, and specificity at specified cutoff points. *p*-values less than 0.05 were considered statistically significant. The Kaplan–Meier curve was used to assess the relationship between OS and PFS over 36 months utilizing the log-rank tests. Univariant and multivariate Cox regression was used to clarify independent prognostic factors. Hazard ratios, as well as a 95% confidence interval, were displayed. The results were judged significant at a *p*-values of 0.05 or less.

## 5. Conclusions

Our findings demonstrate several key advances in B-NHL diagnostics and prognostication. First, the combination analysis of *BAFF* mRNA, *FCRL1* mRNA, and serum LDH levels achieved exceptional diagnostic accuracy with 100% sensitivity and specificity at predefined cutoff points, significantly improving over single-marker approaches. Second, *FCRL1* mRNA expression emerged as a robust independent prognostic indicator, with high expression levels significantly correlating with reduced PFS and OS in Egyptian DLBCL patients receiving R-CHOP therapy. This association remained significant after adjusting for established clinical parameters, suggesting its utility as a novel risk stratification tool.

Conclusively, these findings advance our understanding of DLBCL biology while offering practical tools for clinical management. Integrating these biomarkers into routine clinical practice could significantly enhance diagnostic precision and treatment optimization, particularly for patients receiving R-CHOP therapy.

## Figures and Tables

**Table 1:** Demographic characteristics of the case and control.

		Group				*p*-Value
		Control (N = 40)		DLBCL (N = 60)		
		N	%	N	%	
Gender	Female	23	46.0%	27	54.0%	0.307
	Male	17	34.0%	33	66.0%	
Age	Mean ± SD	41.9 ± 13 40.5, 20		54 ± 15.7 56.5, 23		<0.001
Smoking	No	26	49.1%	27	50.9%	0.06
	Yes	14	29.8%	33	70.2%	

Data presented as n (%) for categorical variables and mean ± SD for continuous variables. Statistical comparisons were performed using the Chi-square test for categorical variables and the independent *t*-test for continuous variables. SD = standard deviation.

**Table 2:** Tumor characteristics in the study participants.

		N	%
**Lugano staging**	IA	19	31.7%
	IB	2	3.3%
	IIA	4	6.7%
	IIB	5	8.3%
	IIIA	5	8.3%
	IIIB	5	8.3%
	IVA	13	21.7%
	IVB	7	11.7%
**Extra-nodal site**	no	45	75.0%
	yes	15	25.0%
**Name of extra-nodal site**	liver	5	33.3%
	lung	4	26.6%
	nasopharynx	1	6.7%
	orbit	1	6.7%
	parapharyngeal mass	1	6.7%
	parotid	1	6.7%
	stomach	1	6.7%
	testicular	1	6.7%
**Performance status**	0	32	53.3%
	1	23	38.3%
	2	5	8.3%
**IPI**	low	28	46.7%
	low intermediate	21	35.0%
	high intermediate	10	16.7%
	high	1	1.7%

**Table 3:** Diagnostic performance of *FCRL 1* and *BAFF* Expressions and LDH serum levels for discriminating cases from the control group.

	Cutoff Point	AUC with 95% CI	*p*-Value	Sensitivity	Specificity	PPV (%)	NPV (%)	Accuracy (%)
*FCRL 1* RQ	2.32	0. 840 (0.75–0.92)	**<0.001**	78%	98%	97.87%	73.58%	85.00%
*BAFF* RQ	1.16	0.976 (0.95–0.99)	**<0.001**	93.3%	85%	90.32%	89.47%	90%
LDH (U/L)	179	0.89(0.82–0.95)	**<0.001**	85%	77%	84.75%	77.50%	81.00%
Combination *FCRL 1* + *BAFF* + LDH		1.000 (1.0–1.0)	**<0.001**	100.0	100.0	100.0%	100.0%	100.0%

**Table 4:** Association between *BAFF* expression, *FCRL1*, and LDH with different clinical criteria.

		*BAFF* RQ		*p*-Value	*FCRL1* RQ		*p*-Value	LDH		*p*-Value
		Mean	SD		Mean	SD				
Gender	Male	2.3	0.9	0.7	5.944	7.661	0.150	578.9	326.7	0.05
	Female	2.1	0.9		3.957	5.907		603.8	475.2	
Smoking	Yes	2	0.8	0.47 *	6.077	7.848	0.124	683.6	378.9	0.9
	No	2.4	1		3.952	5.782		475.9	395.3	
Lugano staging (modified)	IA	2.1	0.8	0.1	8.557	9.007	0.56	172.7	37.3	0.00 *
	IB	1.3	0.1		0.085	0.078		315	14.1	
	IIA	2.2	1		8.003	10.863		365.2	82.2	
	IIB	3.1	1.5		9.036	5.351		484.2	71.9	
	IIIA	2.3	1		12.404	13.299		604.2	26.9	
	IIIB	1.9	0.9		2.478	1.843		773.6	73.5	
	IVA	1.8	0.4		5.649	3.134		998	245.9	
	IVB	2.7	0.9		8.084	9.640		1107.4	304.4	
Extra-nodal site	Yes	2.2	0.8	0.6	6.463	6.688	0.930	423.6	314.6	0.3
	No	2.2	0.9		7.721	8.418		645.6	408.9	
Performance status	0	2.2	0.9	0.1	5.870	7.869	0.06	614.5	382.8	0.6
	1	2	0.8		7.886	6.713		532.4	362.2	
	2	2.9	0.6		15.030	10.952		700	654.3	
IPI	Low	1.9	0.9	0.1	6.5	8	0.158	394.2	274.4	0.00 *
	Low intermediate	2.3	0.8		7.1	6.5		732.1	317.9	
	High intermediate	2.3	0.8		9	10.3		723.4	494.1	
	High	3.7			19.500			1761		
progression	No	2.1	0.9	0.9	6.498	6.818	0.840	642.7	375.4	0.7
	Yes	2.3	0.9		7.895	8.593		492.5	426.2	

**Table 5:** Univariate and multivariate COX regression analysis for the parameters affecting poor outcomes.

	Univariate		Multivariate	
	*p*	HR (LL–UL 95%C.I)	*p*	HR (LL–UL 95%C.I)
Gender (female)	0.471	0.792(0.420–1.493)		
Age	0.109	1.019(0.996–1.043)		
Smoking	0.049 *	1.916(1.001–3.667)	0.063	1.857(0.967–3.565)
Lugano staging	0.142	1.211(0.938–1.562)		
Extra-nodal site	0.404	0.718(0.330–1.563)		
Performance status 1	0.545	0.852(0.507–1.432)		
Performance status 2	0.803	1.065(0.650–1.745)		
IPI (high vs. low)	0.151	1.728(0.819–3.647)		
*FCRL 1* (high expression)	0.037 *	1.966(1.041–3.713)	0.047 *	1.908(1.007–3.613)
*BAFF* (high expression)	0.719	0.891(0.474–1.674)		
LDH (high serum level)	0.359	1.347(0.713–2.542)		

**Table 6:** Log-rank test for overall Survival with *FCRL 1*, *BAFF*, and LDH.

*FCRL 1*	Mean	% End of Study	Log-Rank	
			χ^2^	*p*
Low expression (≤4.95)	34.20	86.7%	4.650 *	0.031 **
High expression (>4.95)	28.87	63.3%		
***BAFF***				
Low expression (≤1.90)	32.519	77.8%	0.222	0.638
High expression (>1.90)	30.727	72.7%		
**LDH**				
Low serum (≤561.50) U/L	31.47	80.0%	0.626	0.429
High serum (>561.50) U/L	31.60	70.0%		

**Table 7:** Log-rank test for progression-free survival with FCR1, BAFF, and LDH.

*FCRL 1*	Mean	% End of Study	Log-Rank	
			χ^2^	*p*
Low expression (≤4.95)	**29.533**	40.0%	4.324	0.038 *
High expression (>4.95)	19.233	30.0%		
***BAFF***				
Low expression (≤1.90)	25.52	31.7%	0.012	0.911
High expression (>1.90)	23.24	36.4%		
**LDH**				
Low serum (≤561.50) U/L	25.17	43.3%	1.583	0.208
High serum (>561.50) U/L	23.37	25.5%		

Log-rank test analysis of progression-free survival stratified by FCRL1, BAFF expression, and LDH levels. Data are presented as mean survival time (months) and percentage of patients progression-free at the end of the study.

## Data Availability

Data supporting this study’s findings will be made available from the corresponding author upon reasonable request.
